# Effects of Moderate Static Magnetic Field on Neural Systems Is a Non-invasive Mechanical Stimulation of the Brain Possible Theoretically?

**DOI:** 10.3389/fnins.2020.00419

**Published:** 2020-05-19

**Authors:** Antonio Hernando, Fernando Galvez, Miguel A. García, Vanesa Soto-León, Carlos Alonso-Bonilla, Juan Aguilar, Antonio Oliviero

**Affiliations:** ^1^Instituto Magnetismo Avanzad, Laboratorio Salvador Velayos, Universidad Complutense de Madrid-Consejo Superior Investigación Cientifica-Administrador Infraestructuras Ferroviarias, Madrid, Spain; ^2^Instituto Madrileños de Estudios Avanzados Nanociencia, Madrid, Spain; ^3^Donostia International Physics Centre, San Sebastián, Spain; ^4^Instituto de Cerámica y Vidrio, Consejo Superior Investigación Cientifica, Madrid, Spain; ^5^Functional Exploration and Neuromodulation of the Nervosus System Investigation Group, Hospital Nacional de Parapléjicos, Servicio de Salud de Castilla la Mancha, Toledo, Spain; ^6^Experimental Neurophysiology, Hospital Nacional de Parapléjicos, Servicio de Salud de Castilla la Mancha, Toledo, Spain; ^7^Hospital Los Madroños, Madrid, Spain

**Keywords:** static magnetic field, Zeeman energy, membrane channels, non-invasive brain stimulation, mechanical stimulation

## Abstract

Static magnetic fields have been shown to induce effects on the human brain. Different experiments seem to support the idea that moderate static magnetic field can exert some influence on the gating processes of the membrane channels. In this article we visit the order of magnitude of the energy magnetic terms associated with moderate applied field (between 10 and 200 milliteslas). It is shown that gradients of the Zeeman energy associated with the inhomogeneous applied fields can induce pressures of the order of 10^–2^Pa. The surface tension generated by the magnetic pressure, on the surface delimiting the brain region subject to relevant field and gradients, is found to range between 10^–1^ and 1 mN⋅m^–1^. These pressures seem to be strong enough to interfere with the elastic and electrostatic energies involved in the channel activation-inactivation-deactivation mechanisms of biological membranes. It has been described that small mechanical force can activate voltage gated potassium channels. Moreover, stretch-activated ion channels are widely described in different biological tissues. Virtually, all these channels can modify their activity if stressed by a sufficient pressure delivered for enough time. We propose mechanical stimulation – possibly not exclusively – as a candidate mechanism how static magnetic field can produce effects in biological systems. It must be emphasized, that such field gradients were not previously proposed as a possible source of neural activity modification.

## Introduction

Static magnetic fields have been shown to induce effects on the human brain (Oliviero et al., 2011; Roberts et al., 2011), and there is evidence of their interference with neuronal function in animals (Rosen and Lubowsky, 1987; McLean et al., 2008; Wu and Dickman, 2012; Aguila et al., 2016). Specifically, applying transcranial static magnetic field stimulation (tSMS) over the human precentral cortex reduces the excitability of the motor cortex (Oliviero et al., 2011; Silbert et al., 2013; Nojima et al., 2015; Arias et al., 2017) and can transiently alter the intracortical inhibitory system (Nojima et al., 2015; Dileone et al., 2018). Moreover, application of tSMS over the visual or parietal cortices produces a focal increase in the power of alpha oscillations, inducing behavioral consequences (Gonzalez-Rosa et al., 2015; Aguila et al., 2016; Carrasco-López et al., 2017).

The brain cells have electric properties, and the connections between brain cells are largely due to an electric coupling. The link between magnetic fields and electricity is well-known. Despite a number of reports about the possibility that static magnetic fields might interfere with the physiological brain functions, a mechanistic explanation of these effects is lacking. In this article, we show that static magnetic fields as those used in references (Rosen and Lubowsky, 1987; McLean et al., 2008; Oliviero et al., 2011; Roberts et al., 2011; Wu and Dickman, 2012; Aguila et al., 2016) produce local variations of the pressure that could be strong enough to modify some biophysical parameters. These biophysical parameter modifications might be responsible – at least in part – for the effects of moderate static magnetic field on human brain.

Up to now, two main streams of studies have been carried out on the possible disturbance of the microscopic biological systems induced by the action of externally applied static magnetic fields. (A) Action on paramagnetic or possible superparamagnetic or ferromagnetic molecules or aggregates (Wheeler et al., 2016) and (B) action on diamagnetic anisotropic aligned macromolecules as membrane lipid bilayers (Rosen, 2003), transmembrane proteins and microtubules. However, a rigorous estimation of the order of magnitude of these effects for applied fields weaker than 1 T indicate that they seem to be negligible for any relevant functional modification (Meister, 2016).

Static fields only can produce Lorentz force on the electric charges in motion and magnetic moments that form the matter. Therefore, the effect of fields on live matter can emerge from the Lorentz force exerted on different charges or moments and can be classified as follows:

a) Ionic currents, only present through the cell membranes. The magnetic field produced by these currents are used in magnetoencephalography observations. Considering a current of intensity *I* that runs along a total length Δ*s*, the magnetic field created by this current would be proportional to the product *I*⋅Δ*s*, which for a neuron is about 10 fA⋅m (10^–14^ A⋅m), and inversely proportional to the square of the distance between the segment Δ*s* and the point where the field is measured. Considering Δ*s* to be 0.1 mm, and, that for a single neuron, the typical presynaptic current ranges between 10^2^ and 10^4^ fA; for instance. From these estimations, it turns out that the Lorentz force exerted by applied static fields weaker than 1 T on any single ionic current flowing during the neural activity is lower than 10^–15^ N (*I* = 10^4^ fA, Δ*s* = 0.1 mm). On the other hand, the electrostatic field acting along the direction perpendicular to the membrane is due to a gradient of 70 mV through 1 nm of membrane thickness. Therefore, this field is approximately of 10^7^ V/m and exerts on the K^+^ ion an electric force of 10^–11^ N which is four orders of magnitude larger than the Lorentz force.
b) Permanent magnetic moments of atomic nuclei that in the case of hydrogen are used in nuclear magnetic resonance diagnosis. The contribution to the macroscopic magnetization of the atomic nuclei is expected to be 10^–3^ times that due to the electronic contribution.
c) Permanent magnetic moments of free radicals, produced during some biological chemical reactions, and molecules and macromolecules containing few paramagnetic atoms, as is the case of deoxyhemoglobine, or macromolecules for which the paramagnetic atoms constitute a large volume fraction and that eventually might behave as superparamagnetic nanoparticles, as is the case of ferritine. Effects on ferritine and Fe atoms contained in proteins have been shown by Meister to be energetically irrelevant as concerns disturbance of the normal biological activity (Meister, 2016). In fact, the Zeeman energy is many orders of magnitude smaller than the energy involved in biological processes.
d) Diamagnetic anisotropy. Tissues, some of them formed by macromolecules showing high anisotropy as is the case of assemblies of parallel oriented uniaxial units, as lipid bi-layers and some proteins as microtubules. Helfrich calculated the elastic effect produced by a magnetic field of 1 T intensity on a single spherical cell (Helfrich, 1973, 1974). Due to the diamagnetic anisotropy of the lipid chains that form its membrane the sphere deforms to an ellipsoid. If the radius of the sphere, when no field is applied, is 1,000 A the difference between the half axes parallel and perpendicular to the field becomes 0.1 A.
e) Artificially introduced magnetic nanoparticles that has been used for generating high magnetic field gradients and membrane stresses modulated by external applied fields (Demir et al., 2015; Lewis, 2016; Tay et al., 2016). As Meister has shown the order of magnitude on the involved energies cannot produce any relevant effect as those claimed in the corresponding publications (Meister, 2016).

Once the aforementioned effects have been initially disregarded as causes of the transcranial static magnetic stimulation, we focus on the macroscopic effect produced by an inhomogeneous magnetic field on a continuous medium with average diamagnetic susceptibility χ.

It is well-known that inhomogeneous magnetic fields exert measurable forces on diamagnetic substances (Hernando and Rojo, 2001). Gouy method, used to determine experimentally the susceptibility of diamagnetic and paramagnetic samples, allows to detect forces of milligrams with field gradients of 1 T/m on 1 cm diameter cylinders. It is obvious then that a macroscopic approach depicted by a diamagnetic medium, with a spatial average susceptibility obtained by the proper procedure from the diamagnetic susceptibility of the components, seems to be an adequate scenario, as concerns order of magnitude of the involved energies and forces, to analyse the possible effects of the magnetic field on the neural activity. This is the basic argument developed along this article.

### Magnetic Susceptibility in Biological Systems

When a large number of atoms and molecules are aggregated forming tissues or cell membranes, they can be treated in terms of a continuum medium and the magnetic moment of the electrons, atoms and molecules are depicted by means of the density of magnetic moment or magnetic moment per unit volume, mass or mole. This density is known as magnetization, ***M***. For linear magnetic materials, it stands that *M* = χ*H*, where the proportionality factor χ is called susceptibility, a dimensionless constant, scalar or tensor that relates the magnetization developed by a substance under the effect of an applied magnetic field (Hernando and Rojo, 2001). According to this definition, χ provides the magnetic moment that appears per unit volume when the sample is subjected to a unit intensity applied field. It is possible to describe the magnetic moment per unit mass induced by an applied field of unit intensity by defining mass susceptibility, χ_m_, which is related to the volume susceptibility through χ_m_ = χ/ρ, with ρ standing for the density of the substance in kg/m^3^ (international system units, SI units). Typical values of diamagnetic mass susceptibility (corresponding to a kilogram of the substance) are, in SI units, −0.91⋅10^−8^ for water, −2.5⋅10^−8^ for hydrogen and −1.71⋅10^−8^ for bismuth.

Another common way of depicting the diamagnetic properties of matter is through the molar susceptibility, that can be obtained by multiplying the mass susceptibility by the molecular weight and by 10^–3^. It is also usual to use the centimeter-gram-second system (CGS unit system), for which the mass susceptibility is expressed in grams and obtained by dividing the value in SI units by −4π⋅10^−3^.

Table 1 summarizes some relevant susceptibility values corresponding to different amino acids and phospholipids. In those proteins containing aromatic chains the susceptibility becomes large due to the benzene-like induced ring currents.

**TABLE 1:** Calculated pressure for different organic media (χ_mol_data extracted from Swift et al., 2008).

Medium	χ_mol_ (10^–6^) (*CGS*)	χ (10^–6^) (*SI*)	*P*_*zH*_ (10^–3^) (*N/m^2^*)	*P*_*zH*_ (10^–2^) (*N/m^2^*)
Alanine	−50	−10	−45.2	−9.60
Glutamic acid	−75	−9.35	−42.2	−2.65
Histidine	−85	−2.96	−13.4	64.4
Isoleucine	−82	−8.13	−36.7	10.2
Tryptophan	−120	−10.3	−46.7	−13
Tyrosine	−115	−10.4	−46.8	−13.3
Lecithin	−70	−1.13	−5.11	83.6
Water	−13	−9.1	−41.1	–

Diamagnetism is the common magnetic behavior of the biological matter, since biological molecules lack permanent magnetic moment and form assemblies that macroscopically behave as diamagnetic media.

In general, the molecular magnetic susceptibility of biological molecules as lipids and proteins is anisotropic. Various diamagnetic biological systems as retinal-rod outer segment (Hong, 1980), lipids (Speyer et al., 1987; Prosser et al., 1998), and chloroplasts (Worcester and Franks, 1976; Pauling, 1979; Sakurai et al., 1980) show magnetic orientation pointing out the existence of diamagnetic anisotropy. For example, a molar susceptibility anisotropy of Δχ_mol_ = −5.36⋅10^−6^, in CGS units, is found for planar peptide groups with resonance between two valence bonds.

The major component of membranes (Speyer et al., 1987) is the phospholipid lecithin, for which its orientation with the long axis perpendicular to the applied field (Δχ_mol_ < 0) has been studied by analyzing the enhancement of the perpendicular edges of the powder lineshapes in the resonance spectra (Worcester and Franks, 1976).

As the cortex is composed of an aqueous medium in which different cells are immersed the local susceptibility changes with a typical fluctuation length of a few nanometers. When trying to find an average susceptibility it is mandatory to define a relevant volume in which the average procedure must be carried out. The fluctuation length of local susceptibility is so short that the forces exerted by a uniform field on the unit volume, due to the susceptibility fluctuations, averages to zero. Thereby, as it concerns force estimation, the average susceptibility could be calculated for the whole cortex volume and considered constant.

### Magnetic Fields in Transcranial Static Magnetic Stimulation and Induced Forces

The magnetic fields, *B* = μ_0_*H*, used in transcranial stimulation are produced by magnets located on the skull. The field gradients∇⁡*H*^2^,and spatial variations of susceptibility, ∇⁡χ, give rise to forces and pressures that act on the neural tissues. Since for the biological materials present in the cortex the susceptibility χ can be treated as a scalar, the force acting on the unit volume is given by Hernando and Rojo (2001):

(1) $$F=-\nabla \left(M\circ B\right)=-\nabla \left(\chi \cdot H\circ \mu_{0}\cdot H\right)=-\mu_{0}\,{\left|H\right|}^{2}\,\nabla \chi -\mu_{0}\,\chi \,\nabla {\left|H\right|}^{2}$$

Please note that ∘ stands for the scalar product between vectors and the |*H*|^2^ components originate from the inner product between *M* and *B* vectors. As discussed above the force given by the term associated with the susceptibility gradient averages to zero. The estimation is reduced to calculate the second term μ_0_∇⁡|*H*|^2^, which could be done by determining the field gradient and considering different reasonable average susceptibilities.

The field distribution as well as its corresponding gradient within the cortex has been calculated by solving the well-known equations depicting the field created by a cylindrical magnet. Moreover, we have measured the field produced by a magnet in air and the results compared with those obtained by calculations to test its reliability.

The values of the susceptibility used for calculations were the lower and upper extremes that contribute to the average value (see Table 1 for further details). The expected average value of the susceptibility should be close to that of water.

Here, we present a novel mechanistic hypothesis of how tSMS induces effects in the biological systems and in human brain. The hypothesis is based on the theoretical effects of the magnetic pressure exerted by a diamagnetic biological medium onto its surroundings when such medium is placed near a static magnetic field gradient. We estimate the order of magnitude of the energy magnetic terms associated with moderate applied magnetic field (between 10 and 200 milliteslas). We show that gradients of the Zeeman energy associated with the inhomogeneous applied fields can induce pressures of the order of 10^–2^ Pa. The surface tension generated by the magnetic pressure, on the surface delimiting the brain region subject to relevant field and gradients, is found to range between 10^–1^ and 1 mN⋅m^–1^. These pressures seem to be strong enough to interfere with the elastic and electrostatic energies involved in the channel activation-inactivation-deactivation mechanisms of biological membranes. Based on our hypothesis and calculations, we propose mechanical stimulation – possibly not exclusively – as a candidate mechanism how static magnetic field can produce effects in biological systems.

## Materials And Methods

The goal of this article is to estimate the magnetic pressure exerted by a diamagnetic biological medium onto its surroundings when such medium is placed near a static magnetic field gradient. In order to obtain a detailed characterization of the magnetic field gradient generated by the considered static source, the software *COMSOL Multiphysics* has been used. This software employs a finite element method to calculate and provide the spatial distribution of the physical parameters of interest for a user-defined model. In this case, the studied system consists of a cylindrical (60 mm diameter, 30 mm height) NdFeB permanent magnet, characterized by a remanent magnetization of *M*_r_ = 1018400*A*/*m* parallel to the long axis, and surrounded by vacuum. Further details of the model and the simulations will be given below.

### Estimation of Zeeman Forces Acting on Biological Tissue

As it has been explained, diamagnetic materials only present a non-zero macroscopic magnetic moment in presence of an external applied magnetic field *H*. Since for these linear materials $\chi =\frac{M}{H}\ll 1$ (and consequently *B*≈μ_0_*H*), the Zeeman energy can be rewritten as:

(2) $$E_{Z\,e\,e\,m\,a\,n}=-\mu_{0}\cdot m\circ H$$

The Zeeman energy per unit volume can be expressed in terms of the magnetization:

$$E_{Z\,e\,e\,m\,a\,n\,V}=-\mu_{0}\cdot M\circ H$$

(3) $$=-\mu_{0}\cdot \left(M_{x}\cdot H_{x}+M_{y}\cdot H_{y}+M_{z}\cdot H_{z}\right)$$

Hence, the force associated with the Zeeman energy per unit volume is given by:

$$F_{V}=-\nabla \cdot E_{Z\,e\,e\,m\,a\,n\,V}=-\mu_{0}\cdot \nabla \left(M\circ H\right)$$

(4) $$=-\mu_{0}\cdot \nabla \left(M_{x}\cdot H_{x}+M_{y}\cdot H_{y}+M_{z}\cdot H_{z}\right)$$

**FIGURE 1:**
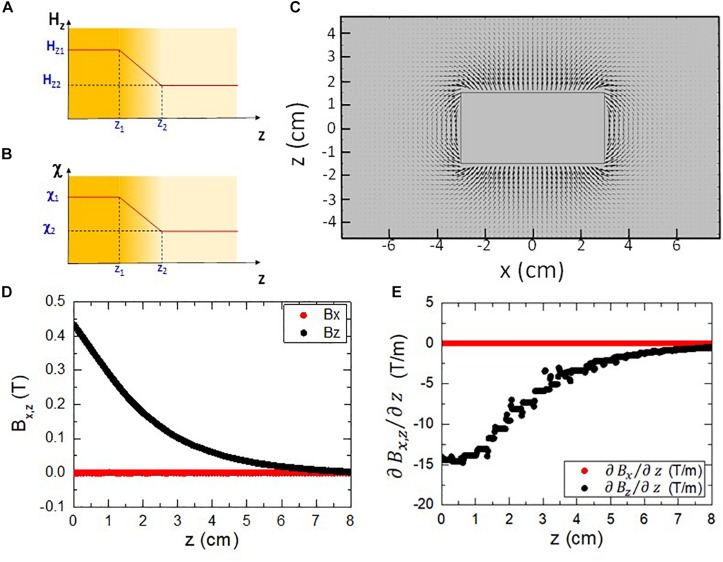
**(A)** Diagram of a spatial region with a magnetic field Hz which varies linearly. **(B)** Diagram of a spatial region with a diamagnetic medium whose susceptibility varies linearly. **(C)** Modeling of the magnetic field around a 60 mm diameter, 30 mm height NdFeB magnet. The area shown corresponds to a XZ cut plane at the center (*y=0*) of the magnet. **(D)** Calculated magnetic field components along a vertical line for the simulated magnet shown in **(C)**. **(E)** Calculated first derivative of the magnetic field along a vertical line for the simulated magnet shown in **(C)**.

Let us consider the case of an isotropic linear diamagnetic material with homogeneous susceptibility$\frac{M_{x}}{H_{x}}=\frac{M_{y}}{H_{y}}=\frac{M_{z}}{H_{z}}=c\,o\,n\,s\,t=\chi$, which occupies a region of the space with thickness Δ*z* = *z*_2_−*z*_1_ where the *z* component of the magnetic field varies linearly Δ*H*_*z*_ = *H*_*z*2_−*H*_*z*1_ as shown in Figure 1A.

First of all, it is important to notice that for this case Equation (4) can be rewritten as:

(5) $$F_{V}=\mu_{0}\cdot \chi \cdot \nabla \left(H_{x}^{2}+H_{y}^{2}+H_{z}^{2}\right)=\mu_{0}\cdot \chi \cdot \nabla {\left|H\right|}^{2}$$

Where $\left|H\right|=\sqrt{H_{x}^{2}+H_{y}^{2}+H_{z}^{2}}$ stands for the modulus of the magnetic field vector *H*. Note that this corresponds to the second term of Equation (1), which is the one dependant on the gradient of the magnetic field.

If we consider that the only field gradient present in this region is along the z-axis direction and varies linearly, the pressure (magnetic force per unit area) exerted by this region over the rest of the material is given by:

$${P_{z}|}_{\chi =c\,o\,n\,s\,t}\equiv P_{z\,\chi }=2\cdot \mu_{0}\cdot \chi \cdot \Delta \,H_{z}\cdot H_{z\,1}+2\cdot \mu_{0}\cdot \chi$$

(6) $$\cdot {\left(\frac{\Delta \,H_{z}}{\Delta \,z}\right)}^{2}\cdot \left(\frac{z_{2}^{2}-z_{1}^{2}}{2}+z_{1}\cdot \Delta \,z\right)$$

The detailed calculation which leads to Equation (6) can be found in the Supplementary Appendix (calculation of magnetic pressure associated with Zeeman energy gradients).

Now, if the case considered is that of a linear isotropic diamagnetic material with magnetic susceptibility that varies linearly in the range Δ*z* = *z*_2_−*z*_1_, with a total variation Δχ = χ_2_−χ_1_ as shown in Figure 1B and under a constant magnetic field, a similar calculation (Supplementary Appendix) demonstrates that the pressure exerted by this region over the rest is given by:

(7) $${P_{z}|}_{H=c\,o\,n\,s\,t}\equiv P_{z\,H}=\mu_{0}\cdot \left(H_{x}^{2}+H_{y}^{2}+H_{z}^{2}\right)$$

### Estimation of the Effective Surface/Volume Under the Magnet Effect

In order to estimate the volume which is affected by the magnetic field created by the cylindrical magnet, and thus the effective surface where the magnetic pressure is being exerted, it must be considered the distribution of |*H*|^2^ both axially and radially.

Once the effective surface has been estimated, it is possible to obtain the surface tension, *σ*, using the Young-Laplace equation:

(8) $$\sigma =\frac{\Delta \,P\cdot R}{2}$$

Where *R* is the radius of curvature of the surface and Δ*P* is the pressure acting on it. As explained below, we will assume for our estimations that Δ*P* = *P*_*z*_, since the contribution from the vertical gradient of ${\left|H\right|}_{z}^{2}$ is much bigger than the radial contribution.

## Results

For this study, the model shown in Figure 3A has been considered. In this model, a 2 mm thick uniform, isotropic medium with magnetic susceptibility χ is placed at a distance of 2 cm from a cylindrical NdFeB permanent magnet (60 mm diameter, 30 mm height). The isotropic diamagnetic medium plays the role of a simplified cortex model, with an average magnetic susceptibility estimated to be that of water (several relevant aminoacids have been considered as examples). This medium is under the effect of the magnetic field gradient created by the magnet, therefore a pressure acts on the “cortex” test model.

In order to obtain a detailed characterization of the magnetic field generated by the considered static source, the software *COMSOL Multiphysics* has been used, as mentioned earlier. The simulations were performed in a simple model consisting on a solid cylindrical magnet (60 mm diameter, 30 mm height) with a remanence of *M*_r_ = 1018400*A*/*m* along the z-axis. This magnet is placed inside an air sphere with radius of 10 cm which is large enough to ensure that the magnetic field distribution near the magnet is not altered by the boundary conditions (i.e., magnetic insulation at the external borders of the model). A meshing with elements of 10^–3^–10^–2^ m was set for the simulations, allowing a 0.05 scaling when necessary for narrow regions.

As expected, the results of the simulation that can be seen in Figure 1C show a distribution of field lines, which run parallel to the long axis at the center of the magnet but get more bent as they approach the edges of the magnet, eventually closing into the opposite face. The strength of the magnetic field, represented by the size of the arrows in Figure 1C, decreases as the distance to the magnet increases. This is the gradient of the magnetic field that is able to induce a local force as it has been pointed out earlier.

As they are required to calculate the induced local forces, the magnetic field components and its spatial derivatives have been evaluated. The region chosen for the study is a vertical line which crosses the center of the magnet (*x*,*y* = 0), since this is the area for which the magnetic field is the strongest. The values for the horizontal (*B*_*x*_) and vertical (*B*_*z*_) components of the magnetic field vector, as well as the first derivative $\frac{\partial }{\partial z}$, are shown in Figures 1D,E. It is worth to note that the horizontal components of both the magnetic field and its first derivative are much lower than their vertical counterparts (thus the conditions assumed for deriving Equation (6) are met, see Supplementary Appendix for further details).

**FIGURE 2:**
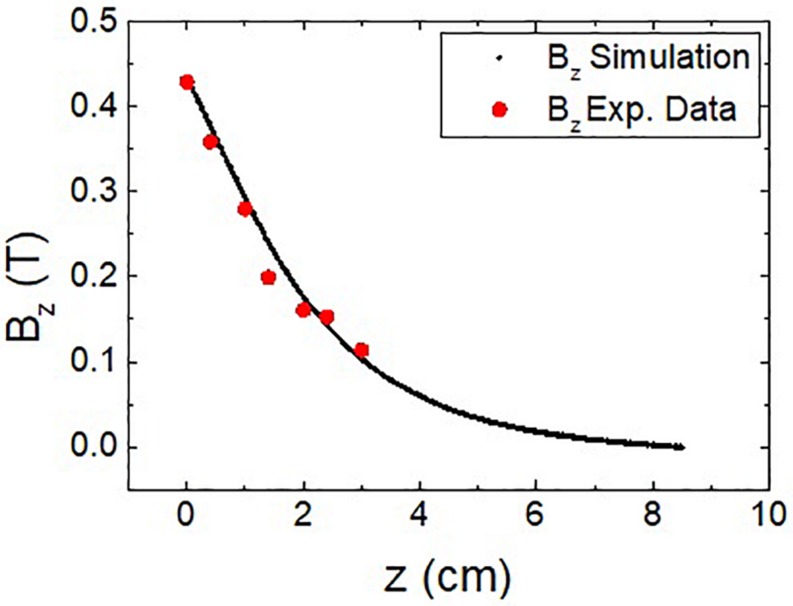
Magnetic field along the vertical axis of a 60 mm diameter, 30 mm height NdFeB magnet: experimental measurements (red dots) vs. simulated field used in the calculations (black dots).

The accuracy of the calculations obtained from the model was checked with an additional experimental test similar to some others described in the literature (Rivadulla et al., 2014). In this test, a conventional Hall probe was used to measure the z component of the magnetic field along the axis of a NdFeB magnet with the same dimensions (60 mm diameter, 30 mm height) than the modeled one. The results of the measurements are plotted and compared to the magnetic field from the simulation in Figure 2. As it can be seen, there is a good agreement between the experimental data and the calculated value, thus proving the validity of the model considered. In this case, the combination of finite element calculations and experimental measurements provides a detailed picture of the analyzed system; alternatively, an analytical approach can also be used to calculate the magnetic field spatial distribution, as shown elsewhere (Caciagli et al., 2018).

**FIGURE 3:**
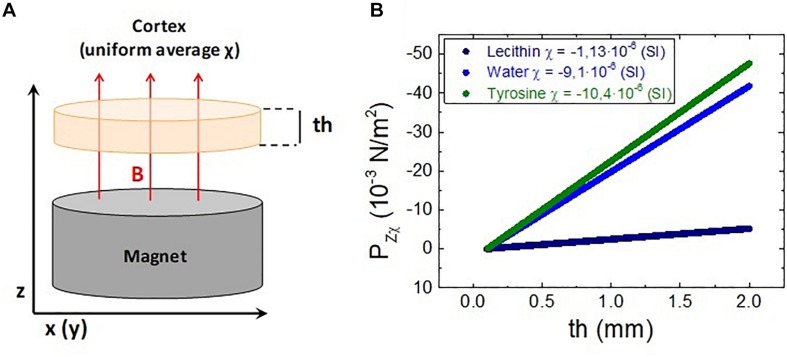
**(A)** Diagram of the model for the magnetic pressure calculations. **(B)** Magnetic pressure exerted on several isotropic diamagnetic media located over the magnet as a function of the medium thickness.

The force per unit volume suffered by an isotropic diamagnetic medium placed at 2 cm over the magnet was calculated using the magnetic field gradient obtained from the simulation. As previously mentioned, the thickness of this medium is 2 mm which approximately accounts for the average thickness of the human cerebral cortex. The magnetic pressure resulting from this force acting on the diamagnetic medium depends on the thickness considered as it is derived from Equation (6). This is shown in Figure 3B using as an example water χ = −9.1⋅10^−6^(*S*.*I*) and two organic molecules with χ = −1.13⋅10^−6^(*S*.*I*) (lecithin) and χ = −10.4⋅10^−6^(*S*.*I*) (tyrosine). When considering the full thickness (2 mm)o f the medium, the total magnetic pressure *P*_*zχ*_ exerted on the cortex model is obtained. The same calculation has been performed for several diamagnetic media, each one composed by a uniform distribution of a single molecule. Note that for the real scenario, a uniform average susceptibility composed from those of these different components should be used. The results obtained are summarized in Table 1.

Regarding the second scenario considered, which is an interface with a linear variation of susceptibility Δχ but under constant magnetic field, the values of *P*_*zH*_ have been calculated and summarized also in Table 1. Equation (7) has been used for this calculation, introducing the value of the field from the simulation at *z* = 2*cm* and considering that the medium of interest (χ_*2*_) is an organic material surrounded by extracellular or intracellular liquid whose susceptibility (χ_*1*_ has been assumed to be approximately that of water [χ = −9.1⋅10^−6^(*S*.*I*)].

**FIGURE 4:**
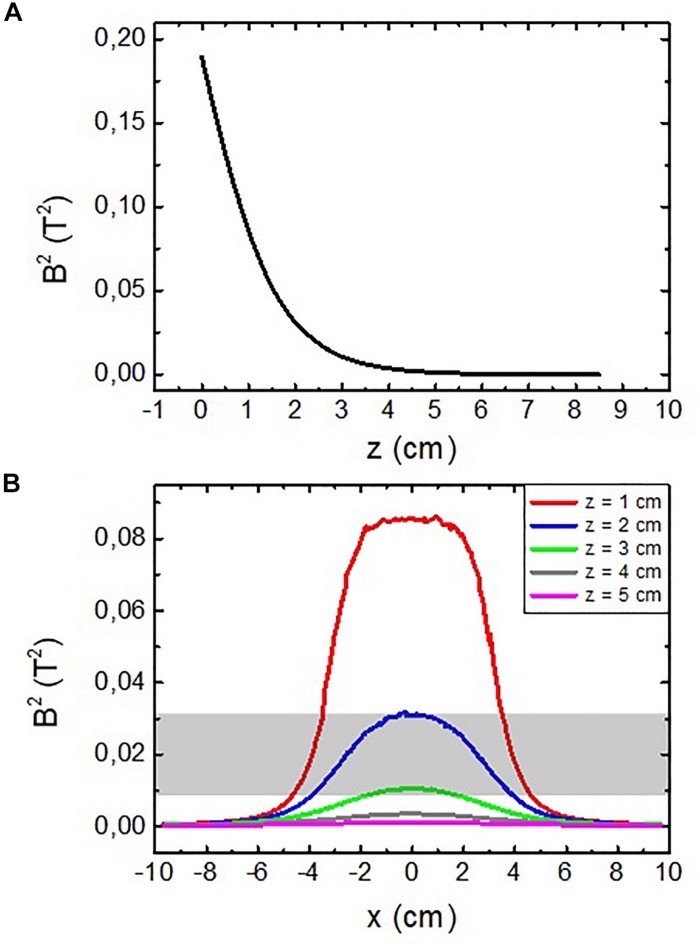
Calculated squared modulus of the magnetic field vector |*B*^2^| for **(A)** axial direction of the system [(*x*,*y*) = (0,0)] and **(B)** radial direction of the system at different separations from the magnet. The darkened area represents the estimated position of the cortex from the magnet surface.

Finally, the effective surface has also been estimated using the value of *B*^2^ obtained from the simulations, as shown in Figure 4.

From Figure 4A the axial limit can be approximately set at *z* = 3 cm, since *B*^2^ has fallen to ∼6% and the gradient of *B*^2^ to ∼10% of its respective maximum values. With an analogous reasoning, the axial limit of the affected volume can be set from Figure 4B at a distance of *r* = 3 cm (note that r can be either the x or y Cartesian coordinate due to the symmetry of the system). Following these estimations, we can consider that the surface affected by the magnetic pressure has a radius of curvature of ∼3 cm.

Introducing in Equation (8) the estimated radius as well as the values of the pressure presented in Table 1, it is possible to obtain the corresponding values for the surface tension. These values are summarized in Table 2.

**TABLE 2:** Calculated surface tension for different organic media with a radius of curvature *R* = 3 cm.

Medium	*P*_*zχ*_ (10^–3^) (*N/m*^2^)	*σ* (10^–1^) (*mN/m*)
Alanine	−45.2	−6.78
Glutamic acid	−42.2	−6.33
Histidine	−13.4	−2.01
Isoleucine	−36.7	−5.51
Tryptophan	−46.7	−7.01
Tyrosine	−46.8	−7.02
Lecithin	−5.11	−7.67
Water	−41.1	−6.15

## Discussion

It is well-established that through the influence of the surface tension on the structure of the channel proteins, the membrane deformations and modification of its surface tension affect the kinetics of voltage channel gating mechanism. The influence of pressure on the elastic energy of any membrane has been described and thoroughly analyzed in an extensive literature (Zhong-Can and Helfrich, 1987; García-Sáez et al., 2007; Phillips et al., 2009; Baumgart et al., 2011). Please note that in the literature it is also frequent to see use of the term “line tension” instead of “surface tension”; as far as it concerns this article, both terms can be considered equivalent.

Since 1984 it is known that some Na^+^ and K^+^ voltage channels are mechanosensitive even though they are not mechanosensors (Conti et al., 1984; Gu et al., 2001; Sigg, 2014). This effect has been seen directly by bilayer tension in bacterial channel as well as by the two-pore domain K^+^ channels Trek-1 and TRAAK. In particular *Shaker-IR*, voltage-gated of K^+^ (Gu et al., 2001), has been shown in oocytes to exhibit a robust mechanosensitive behavior. The complex structure of this type of channels in humans is now well-established (Brohawn et al., 2012), though a microscopic detailed knowledge of the physical governing its gating has not yet been satisfactorily reached (Conti et al., 1984; Sigg, 2014). According to the experiments reported in the literature, for different cells and voltages the threshold surface tension required to induce stretch-activation or inactivation should generate ranges between 2 and 15 mN/m (Goulian et al., 1998; Gu et al., 2001; Yang et al., 2011; Brohawn et al., 2012; Peyronnet et al., 2014; Cox et al., 2016). However, it is important to remark that the open probability P_0_ of these pores, that depend on voltage, are affected strongly by changes of a few per cent of the surface tension around its threshold value (Goulian et al., 1998; Gu et al., 2001; Peyronnet et al., 2014; Cox et al., 2016). The surface tension generated by the magnet at the interphases between field affected and unaffected regions has been found to be close to 2⋅10^–1^ mN/m that is in the range comprised between the 1 and 10% of the usual thresholds of mechanosensitive channels. Therefore, the effect of the field gradient could modify between 1 and 10% the activation or inactivation probabilities in those channels located at the interphase boundaries that, given their dimensions, should include a huge number of neurons and, thereby a considerable percentage of them. As stated above, the calculation of the tension has been performed by considering a uniform susceptibility through the whole cortex. Estimation of the local tension in a given neuron membrane embedded in this medium would require further order of approximation by taking into account susceptibility fluctuations around its average value. Anyway, local tension must deviate from the average tension no more than the limits defined by the distribution of individual values of the susceptibility for the different components of the membrane and extra and intra cellular media. However, what it is here emphasized is that the average tension of the medium in which neurons are embedded noticeably changes under the action of the external field. Such modification of the average tension should affect the local membrane stress and consequently could bias its channels gating kinetics.

The mechanisms how tSMS affects the cortex are largely unknown. It can be obvious to think that field gradients and susceptibility fluctuations can interfere with the electric properties of neural structures. On the other hand, these field gradients and spatial variations of susceptibility give rise to forces and pressures acting on the neural tissues. We calculated these forces and pressures. The Zeeman energy associated with the inhomogeneous applied fields can induce through the cortex pressures of the order of 10^–2^ Pa. We do not know if this is enough to produce the behavioral and neurophysiological effects of moderate static magnetic field transcranial application. Hitherto, it is largely enough to produce effects on neural structure like the inner hair cells of ear (as an example of mechanosensor). For instance, the threshold pressure for inner hair cells of ear sensitivity is 10^–5^ Pa (Hudspeth et al., 2000). We are aware that no specific mechanosensors have been described in the brain. On the other hand, it has been described that small mechanical force derived from membrane tension can activate voltage gated potassium channels (Ranade et al., 2015), and probably other ion channels. Finally, stretch-activated ion channels are widely described in different biological tissues, from muscle to primary sensory neurons due to lipid bilayer tension (Anishkin et al., 2014). Virtually, all these channels can modify their activity if stressed by a sufficient pressure delivered for enough time.

At least 10 min of tSMS seems to be necessary to induce long lasting cortical effects (e.g., 1 min tSMS over the motor cortex has no effects on cortical excitability (Oliviero et al., 2011). Thus, it is conceivable that, if applied for enough time (e.g., minutes), the generated pressures are sufficient to modify the normal physiological function of the cortex via a mechanic alteration of excitability in neural cells and/or circuits due to structural modification of channels (and receptors).

We would like to underline that when tSMS is applied over a moving structure (e.g., the human brain moves in relation to the heartbeat) the pressure induced by the static magnetic field will interfere with the pressure of the moving structure. The resulting features from the interplay between both processes acting simultaneously have yet to be explored.

We propose that a mechanical mechanism may contribute to the long-lasting effects of the tSMS over the cortex. Hitherto, this is not necessary the unique mechanism that can determine the neurophyological effects. During tSMS, the brain and arteries (and the neural tissues near them) are moving within a magnetic field, so electrical currents are generated. This means that a repetitive electrical stimulation is interfering with the neural cell functions. Moreover, we cannot exclude other mechanisms causing the tSMS effects on cortical excitability by acting directly or indirectly on the nervous system. Future studies on isolated preparation (e.g., slices) or on single neurons (e.g., isolated or cultured) may clarify the real importance of each of these mechanisms.

In summary, it has been shown that the magnetic field gradient created by a permanent magnet is able to exert pressures on different homogeneous biological media which induce surface tensions whose strength can alter substantially the gating probability of mechanosensitive channels.

The mechanisms how static magnetic fields modulate cortical activity are largely unknown. We suggest that mechanical stimulation – may be not exclusively – is a possible mechanism how static magnetic field can produce effects in biological systems. These effects are possibly due to the modification of channel (and receptor) characteristics caused by the persistent pressure produced by the static magnetic field.

## Data Availability Statement

The datasets generated for this study are available on request to the corresponding author.

## Author Contributions

All authors listed have made a substantial, direct and intellectual contribution to the work, and approved it for publication.

## Conflict of Interest

AO and JA were cofounders of the company Neurek SL, which is a spinoff of the Foundation of the Hospital Nacional de Parapléjicos. AO and JA were inventors listed on the following patents: P201030610 and PCT/ES2011/070290 (patent abandoned). The remaining authors declare that the research was conducted in the absence of any commercial or financial relationships that could be construed as a potential conflict of interest.

## References

[B1] Aguila J. Cudeiro J. Rivadulla C. (2016). Effects of Static magnetic fields on the visual cortex: reversible visual deficits and reduction of neuronal activity. *Cereb. Cortex* 26 628–638. 10.1093/cercor/bhu228 25260705

[B2] Anishkin A. Loukin S. H. Teng J. Kung C. (2014). Feeling the hidden mechanical forces in lipid bilayer is an original sense. *Proc. Natl. Acad. Sci. U.S.A.* 111 7898–7905. 10.1073/pnas.1313364111 24850861 PMC4050596

[B3] Arias P. Adán-Arcay L. Puerta-Catoira B. Madrid A. Cudeiro J. (2017). Transcranial static magnetic field stimulation of M1 reduces corticospinal excitability without distorting sensorimotor integration in humans. *Brain Stimul.* 10 340–342. 10.1016/j.brs.2017.01.002 28094125

[B4] Baumgart T. Capraro B. R. Zhu C. Das S. L. (2011). Thermodynamics and mechanics of membrane curvature generation and sensing by proteins and lipids. *Annu. Rev. Phys. Chem.* 62 483–506. 10.1146/annurev.physchem.012809.103450 21219150 PMC4205088

[B5] Brohawn S. G. del Mármol J. MacKinnon R. (2012). Crystal structure of the human K2P TRAAK, a lipid- and mechano-sensitive K+ ion channel. *Science* 335 436–441. 10.1126/science.1213808 22282805 PMC3329120

[B6] Caciagli A. Baars R. J. Philipse A. P. Kuipers B. W. M. (2018). Exact expression for the magnetic field of a finite cylinder with arbitrary uniform magnetization. *J. Magn. Magn. Mater.* 456 423–432. 10.1016/j.jmmm.2018.02.003

[B7] Carrasco-López C. Soto-León V. Céspedes V. Profice P. Strange B. A. Foffani G. (2017). Static magnetic field stimulation over parietal cortex enhances somatosensory detection in humans. *J. Neurosci.* 37 3840–3847. 10.1523/JNEUROSCI.2123-16.2017 28280254 PMC6596712

[B8] Conti F. Inoue I. Kukita F. Stühmer W. (1984). Pressure dependence of sodium gating currents in the squid giant axon. *Eur. Biophys. J.* 11 137–147. 10.1007/bf00276629 6100544

[B9] Cox C. D. Bae C. Ziegler L. Hartley S. Nikolova-Krstevski V. Rohde P. R. (2016). Removal of the mechanoprotective influence of the cytoskeleton reveals PIEZO1 is gated by bilayer tension. *Nat. Commun.* 7:10366. 10.1038/ncomms10366 26785635 PMC4735864

[B10] Demir S. Jeon I.-R. Long J. R. David Harris T. (2015). Radical ligand-containing single-molecule magnets. *Coord. Chem. Rev.* 289–290 149–176. 10.1016/j.ccr.2014.10.012

[B11] Dileone M. Mordillo-Mateos L. Oliviero A. Foffani G. (2018). Long-lasting effects of transcranial static magnetic field stimulation on motor cortex excitability. *Brain Stimul.* 11 676–688. 10.1016/j.brs.2018.02.005 29500043

[B12] García-Sáez A. J. Chiantia S. Schwille P. (2007). Effect of line tension on the lateral organization of lipid membranes. *J. Biol. Chem.* 282 33537–33544. 10.1074/jbc.M706162200 17848582

[B13] Gonzalez-Rosa J. J. Soto-Leon V. Real P. Carrasco-Lopez C. Foffani G. Strange B. A. (2015). Static magnetic field stimulation over the visual cortex increases alpha oscillations and slows visual search in humans. *J. Neurosci.* 35 9182–9193. 10.1523/JNEUROSCI.4232-14.2015 26085640 PMC6605156

[B14] Goulian M. Mesquita O. N. Fygenson D. K. Nielsen C. Andersen O. S. Libchaber A. (1998). Gramicidin channel kinetics under tension. *Biophys. J.* 74 328–337. 10.1016/S0006-3495(98)77790-2 9449333 PMC1299385

[B15] Gu C. X. Juranka P. F. Morris C. E. (2001). Stretch-activation and stretch-inactivation of Shaker-IR, a voltage-gated K+ channel. *Biophys. J.* 80 2678–2693. 10.1016/S0006-3495(01)76237-6 11371444 PMC1301455

[B16] Helfrich W. (1973). Elastic properties of lipid bilayers: theory and possible experiments. *Z. Naturforsch. C.* 28 693–703. 10.1515/znc-1973-11-1209 4273690

[B17] Helfrich W. (1974). Blocked lipid exchange in bilayers and its possible influence on the shape of vesicles. *Z Naturforsch C Biosci* 29C 510–515. 10.1515/znc-1974-9-1010 4278012

[B18] Hernando A. Rojo J. M. (2001). *Fiìsica de los Materiales Magneìticos.* Madrid: Siìntesis.

[B19] Hong F. T. (1980). Magnetic anisotropy of the visual pigment rhodopsin. *Biophys. J.* 29 343–346. 10.1016/S0006-3495(80)85138-1 7260258 PMC1328703

[B20] Hudspeth A. J. Choe Y. Mehta A. D. Martin P. (2000). Putting ion channels to work: mechanoelectrical transduction, adaptation, and amplification by hair cells. *Proc. Natl. Acad. Sci. U.S.A.* 97 11765–11772. 10.1073/pnas.97.22.11765 11050207 PMC34347

[B21] Lewis S. (2016). Magnetic manipulation. *Nat. Rev. Neurosci.* 17 263–263. 10.1038/nrn.2016.42 27040906

[B22] McLean M. J. Engström S. Qinkun Z. Spankovich C. Polley D. B. Polley D. (2008). Effects of a static magnetic field on audiogenic seizures in black Swiss mice. *Epilepsy Res.* 80 119–131. 10.1016/j.eplepsyres.2008.03.022 18541409

[B23] Meister M. (2016). Physical limits to magnetogenetics. *eLife* 5:e017210. 10.7554/eLife.17210 27529126 PMC5016093

[B24] Nojima I. Koganemaru S. Fukuyama H. Mima T. (2015). Static magnetic field can transiently alter the human intracortical inhibitory system. *Clin. Neurophysiol.* 126 2314–2319. 10.1016/j.clinph.2015.01.030 25792074

[B25] Oliviero A. Mordillo-Mateos L. Arias P. Panyavin I. Foffani G. Aguilar J. (2011). Transcranial static magnetic field stimulation of the human motor cortex. *J. Physiol.* 589 4949–4958. 10.1113/jphysiol.2011.211953 21807616 PMC3224885

[B26] Pauling L. (1979). Diamagnetic anisotropy of the peptide group. *Proc. Natl. Acad. Sci. U.S.A.* 76 2293–2294. 10.1073/pnas.76.5.2293 287071 PMC383585

[B27] Peyronnet R. Tran D. Girault T. Frachisse J. M. (2014). Mechanosensitive channels: feeling tension in a world under pressure. *Front. Plant Sci.* 5:558. 10.3389/fpls.2014.00558 25374575 PMC4204436

[B28] Phillips R. Ursell T. Wiggins P. Sens P. (2009). Emerging roles for lipids in shaping membrane-protein function. *Nature* 459 379–385. 10.1038/nature08147 19458714 PMC3169427

[B29] Prosser R. S. Hwang J. S. Vold R. R. (1998). Magnetically aligned phospholipid bilayers with positive ordering: a new model membrane system. *Biophys. J.* 74 2405–2418. 10.1016/S0006-3495(98)77949-4 9591667 PMC1299583

[B30] Ranade S. S. Syeda R. Patapoutian A. (2015). Mechanically activated ion channels. *Neuron* 87 1162–1179. 10.1016/j.neuron.2015.08.032 26402601 PMC4582600

[B31] Rivadulla C. Foffani G. Oliviero A. (2014). Magnetic field strength and reproducibility of neodymium magnets useful for transcranial static magnetic field stimulation of the human cortex. *Neuromodulation Technol. Neural Interface* 17 438–442. 10.1111/ner.12125 24125470

[B32] Roberts D. C. Marcelli V. Gillen J. S. Carey J. P. Della Santina C. C. Zee D. S. (2011). MRI magnetic field stimulates rotational sensors of the brain. *Curr. Biol.* 21 1635–1640. 10.1016/j.cub.2011.08.029 21945276 PMC3379966

[B33] Rosen A. D. (2003). Mechanism of action of moderate-intensity static magnetic fields on biological systems. *Cell Biochem. Biophys.* 39 163–173. 10.1385/CBB 39:163. 14515021

[B34] Rosen A. D. Lubowsky J. (1987). Magnetic field influence on central nervous system function. *Exp. Neurol.* 95 679–687. 10.1016/0014-4886(87)90308-6 3817086

[B35] Sakurai I. Sakurai S. Sakurai T. Seto T. Ikegami A. Iwayanagi S. (1980). Electron diffraction study on single crystals of l-type and dl-type lecithins. *Chem. Phys. Lipids* 26 41–48. 10.1016/0009-3084(80)90009-2

[B36] Sigg D. (2014). Modeling ion channels: past, present, and future. *J. Gen. Physiol.* 144 7–26. 10.1085/jgp.201311130 24935742 PMC4076515

[B37] Silbert B. I. Pevcic D. D. Patterson H. I. Windnagel K. A. Thickbroom G. W. (2013). Inverse correlation between resting motor threshold and corticomotor excitability after static magnetic stimulation of human motor cortex. *Brain Stimul.* 6 817–820. 10.1016/j.brs.2013.03.007 23598254

[B38] Speyer J. B. Sripada P. K. Das Gupta S. K. Shipley G. G. Griffin R. G. (1987). Magnetic orientation of sphingomyelin-lecithin bilayers. *Biophys. J.* 51 687–691. 10.1016/S0006-3495(87)83394-5 3580492 PMC1329941

[B39] Swift J. C. Pajerowski D. M. Meisel M. W. (2008). *Magnetic Susceptibility of L-Amino Acids in Solid State at High Magnetic Fields.* Available online at: http://www.phys.ufl.edu/REU/2008/reports/swift.pdf (accessed September 2, 2019).

[B40] Tay A. Kunze A. Murray C. Di Carlo D. (2016). Induction of calcium influx in cortical neural networks by nanomagnetic forces. *ACS Nano* 10 2331–2341. 10.1021/acsnano.5b07118 26805612

[B41] Wheeler M. A. Smith C. J. Ottolini M. Barker B. S. Purohit A. M. Grippo R. M. (2016). Genetically targeted magnetic control of the nervous system. *Nat. Neurosci.* 19 756–761. 10.1038/nn.4265 26950006 PMC4846560

[B42] Worcester D. L. Franks N. P. (1976). Structural analysis of hydrated egg lecithin and cholesterol bilayers II. Neutron diffraction. *J. Mol. Biol.* 100 359–378. 10.1016/S0022-2836(76)80068-X 943549

[B43] Wu L.-Q. Dickman J. D. (2012). Neural correlates of a magnetic sense. *Science* 336 1054–1057. 10.1126/science.1216567 22539554

[B44] Yang Y. Yan Y. Zou X. Zhang C. Zhang H. Xu Y. (2011). Static magnetic field modulates rhythmic activities of a cluster of large local interneurons in *Drosophila* antennal lobe. *J. Neurophysiol.* 106 2127–2135. 10.1152/jn.00067.2011 21775714

[B45] Zhong-Can O. Y. Helfrich W. (1987). Instability and deformation of a spherical vesicle by pressure. *Phys. Rev. Lett.* 59 2486–2488. 10.1103/PhysRevLett.59.2486 10035563

