# Supplementary material for: Effects of Moderate Static Magnetic Field on Neural Systems Is a Non-invasive Mechanical Stimulation of the Brain Possible Theoretically?
Source: Front Neurosci. 2020 May 19;14:419. doi: 10.3389/fnins.2020.00419 (PMC7248270; doi:10.3389/fnins.2020.00419)
Supplement: Supplementary file 1 [file Data_Sheet_1.docx]

**Appendix**

**Calculation of magnetic pressure associated with Zeeman energy gradients**

*(Please note that in the equations presented below, the dot symbol (·) will be used for numerical multiplications while the open dot symbol (*$\circ$*) will be used for the scalar product between vectors)*

The Zeeman energy per unit volume stored in a diamagnetic or paramagnetic material is given by:

$E_{V}=-\mu_{0}\cdot H\circ M=-\mu_{0}\cdot\left( M_{x}\cdot H_{x}+M_{y}\cdot H_{y}+M_{z}\cdot H_{z} \right)$ Eq. (1)

So, the force per unit volume will be:

$F_{V}=-\nabla\cdot E_{V}=\mu_{0}\cdot\nabla\left( H\circ M \right)=\mu_{0}\cdot\nabla\left( M_{x}\cdot H_{x}+M_{y}\cdot H_{y}+M_{z}\cdot H_{z} \right)$ Eq. (2)

$=\mu_{0}\cdot\left( \frac{\partial}{\partial x}\left( M_{x}\cdot H_{x}+M_{y}\cdot H_{y}+M_{z}\cdot H_{z} \right),\frac{\partial}{\partial y}\left( M_{x}\cdot H_{x}+M_{y}\cdot H_{y}+M_{z}\cdot H_{z} \right),\frac{\partial}{\partial z}\left( M_{x}\cdot H_{x}+M_{y}\cdot H_{y}+M_{z}\cdot H_{z} \right) \right)$ Eq. (3)

Let us consider two cases:

1. **A isotropic diamagnetic material with uniform susceptibility in presence of a non-uniform applied magnetic field**

$\frac{M_{x}}{H_{x}}=\frac{M_{y}}{H_{y}}=\frac{M_{z}}{H_{z}}=\chi=cte$

For this case, the force per unit volume

$F_{V}=\mu_{0}\cdot\left( \frac{\partial}{\partial x}\left( \chi\cdot H_{x}^{2}+\chi\cdot H_{y}^{2}+\chi\cdot H_{z}^{2} \right),\frac{\partial}{\partial y}\left( \chi\cdot H_{x}^{2}+\chi\cdot H_{y}^{2}+\chi\cdot H_{z}^{2} \right),\frac{\partial}{\partial z}\left( \chi\cdot H_{x}^{2}+\chi\cdot H_{y}^{2}+\chi\cdot H_{z}^{2} \right) \right)$ Eq. (4)

Now, for a region of the space where only the H_Z_ component varies when moving along z axis, being this variation linear: $H_{x}=cte; H_{y}=cte; H_{z}=H_{z1}+\frac{z-z_{1}}{z_{2}-z_{1}}\cdot(H_{z2}-H_{z1}$)

The only derivative non-vanishing from eq. 4 will be:

$\frac{\partial}{\partial z}\left( \chi\cdot H_{z}^{2} \right)=2\cdot\chi\cdot H_{z}\cdot\frac{(H_{z2}-H_{z1})}{z_{2}-z_{1}}$ Eq. (5)

Then, the force per unit volume will be:

$F_{V}=\mu_{0}\cdot\left( 0,0,2\cdot\chi\cdot H_{z}\cdot\frac{(H_{z2}-H_{z1})}{z_{2}-z_{1}} \right)$ Eq. (6)

And the pressure exerted by this region over the rest of the materials will be:

$P=\int_{z1}^{z2} F_{V}\cdot dz$ Eq. (7)

So,

$P=\int_{z1}^{z2} F_{V}\cdot dz=\left( 0,0,\int_{z1}^{z2} \mu_{0}\cdot2\cdot\chi\cdot H_{z}\cdot\frac{(H_{z2}-H_{z1})}{z_{2}-z_{1}}dz \right)$ Eq. (8)

Pressure only has z component that is given by:

$${{P_{z}|}_{\chi=const}\equiv P_{z\chi}=}_{z}=\int_{z1}^{z2} \mu_{0}\cdot2\cdot\chi\cdot H_{z}\cdot\frac{(H_{z2}-H_{z1})}{z_{2}-z_{1}}dz=\int_{z1}^{z2} \mu_{0}\cdot2\cdot\chi\cdot\left( H_{z1}+\frac{z-z_{1}}{z_{2}-z_{1}}\cdot(H_{z2}-H_{z1}) \right)\cdot\frac{(H_{z2}-H_{z1})}{z_{2}-z_{1}}dz$$

=$2\cdot\mu_{0}\cdot\chi\cdot\frac{(H_{z2}-H_{z1})}{z_{2}-z_{1}}\cdot\left[ \left( H_{z1}\cdot z+\frac{\frac{z^{2}}{2}-z_{1}\cdot z}{z_{2}-z_{1}}\cdot(H_{z2}-H_{z1}) \right) \right]_{z1}^{z2}$=

$$=2\cdot\mu_{0}\cdot\chi\cdot\frac{(H_{z2}-H_{z1})}{z_{2}-z_{1}}\cdot\left[ H_{z1}\cdot\left( z_{2}-z_{1} \right)+\frac{(H_{z2}-H_{z1})}{z_{2}-z_{1}}\cdot\left( \frac{z_{2}^{2}-z_{1}^{2}}{2}+z_{1}\cdot\left( z_{2}-z_{1} \right) \right) \right]$$

$P_{z\chi}=2\cdot\mu_{0}\cdot\chi\cdot\left( H_{z2}-H_{z1} \right)\cdot H_{z1}+2\cdot\mu_{0}\cdot\chi\cdot\left( \frac{(H_{z2}-H_{z1})}{z_{2}-z_{1}} \right)^{2}\cdot\left( \frac{z_{2}^{2}-z_{1}^{2}}{2}+z_{1}\cdot\left( z_{2}-z_{1} \right) \right)$ Eq. (9)

Naming $\Delta H_{z}=H_{z2}-H_{z1}; \Delta z$=$z_{2}-z_{1}$ we have

$P_{z\chi}=2\cdot\mu_{0}\cdot\chi\cdot\Delta H_{z}\cdot H_{z1}+2\cdot\mu_{0}\cdot\chi\cdot\left( \frac{\Delta H_{z}}{\Delta z} \right)^{2}\cdot\left( \frac{z_{2}^{2}-z_{1}^{2}}{2}+z_{1}\cdot\Delta z \right)$ Eq. (10)

1. **A isotropic diamagnetic material with non-uniform susceptibility in a uniform magnetic field:**

$\frac{M_{x}}{H_{x}}=\frac{M_{y}}{H_{y}}=\frac{M_{z}}{H_{z}}=\chi=\chi\left( \vec{r} \right)$

Considering equation (4) and assuming that magnetic susceptibility has a linear variation only when moving along z direction and in a region between z_1_ and z_2_ as:

$\chi=\chi_{1}+\frac{z-z_{1}}{z_{2}-z_{1}}\cdot(\chi_{2}-\chi_{1}$); Eq. (11)

Since H is uniform in this region, eq. (4) becomes:

$F_{V}=\mu_{0}\cdot\left( 0,0,\left( H_{x}^{2}+H_{y}^{2}+H_{z}^{2} \right)\frac{\partial\chi}{\partial z} \right)=\left( H_{x}^{2}+H_{y}^{2}+H_{z}^{2} \right)\left( 0,0,\frac{(\chi_{2}-\chi_{1})}{z_{2}-z_{1}}\cdot\right)$ Eq. (12)

And the pressure exerted by this region over the rest of the material is given by:

$P=\int_{z1}^{z2} F_{V}\cdot dz$=$\left( 0,0,\mu_{0}\cdot\left( H_{x}^{2}+H_{y}^{2}+H_{z}^{2} \right)\cdot\int_{z1}^{z2} \frac{(\chi_{2}-\chi_{1})}{z_{2}-z_{1}}\cdot dz \right)$ ; Eq. (13)

Again, we only have pressure along z direction and it is:

$${P_{z}|}_{H=const}\equiv P_{zH}=\int_{z1}^{z2} \mu_{0}\left( H_{x}^{2}+H_{y}^{2}+H_{z}^{2} \right)\frac{(\chi_{2}-\chi_{1})}{z_{2}-z_{1}}\cdot dz=\mu_{0}\cdot\left( H_{x}^{2}+H_{y}^{2}+H_{z}^{2} \right)\cdot\frac{(\chi_{2}-\chi_{1})}{z_{2}-z_{1}}\left[ z \right]_{z1}^{z2}$$

$$P_{zH}=\mu_{0}\cdot\left( H_{x}^{2}+H_{y}^{2}+H_{z}^{2} \right)\cdot\left( \chi_{2}-\chi_{1} \right) Eq. (14)$$
